# Effectiveness and tolerability of lacosamide in children with drug resistant epilepsy

**DOI:** 10.1016/j.ebr.2022.100574

**Published:** 2022-11-14

**Authors:** J.T. Driessen, E.A. Wammes–van der Heijden, P. Verschuure, K.C.F.M. Fasen, M.W.A. Teunissen, H.J.M. Majoie

**Affiliations:** a Department of Research and Development, Epilepsy Centre Kempenhaeghe, Post address: Sterkselseweg 65, 5591 VE Heeze, The Netherlands; b School of Health Professions Education, Faculty of Health, Medicine and Life Sciences, Maastricht University, Maastricht, The Netherlands; c MHeNS, School for Mental Health and Neuroscience, Department of Psychiatry and Neuropsychology, Maastricht University Medical Centre, Maastricht, The Netherlands; d Department of Clinical Pharmacy, Anna Hospital, Geldrop, The Netherlands; e Department of Clinical Pharmacy, VieCuri Medical Centre, Venlo, The Netherlands; f Laboratory for Clinical Chemistry and Pharmacology, Epilepsy Centre Kempenhaeghe, Heeze, The Netherlands

**Keywords:** Lacosamide, Refractory Epilepsy, Effectiveness, Side-effects, Paediatric

## Abstract

We retrospectively studied the efficacy and tolerability of lacosamide (LCM) in children with drug-resistant epilepsy in a tertiary care centre in the Netherlands, from 2013 till 2019, with a follow-up of two years. 79 children, aged < 18 years, were included. Retention rate, effectiveness, reason for termination, and side-effects were analysed. Furthermore, prognostic variables for discontinuation as well as the incidence of side-effects were determined. The LCM retention rate and effectiveness of response were analysed at three, twelve and twenty-four months. The retention rate of LCM was respectively 89.9 %, 68.4 % and 54.4 %. LCM gave an effective response in 60.5 %, 67.9 % and 71.4 % of the participants who were still using LCM at the three follow-up periods. Lack of efficacy was most frequently reported as a reason for discontinuation (58.3 %). Side-effects occurred in 50.6 % of the patients, somnolence (18.2 %) being the most common, followed by behavioural changes (15.6 %), headache (9.1 %) and dizziness (9.1 %). Use of ≥ 1 sodium channel blocker (SCB) was associated with an increased risk (OR = 4.038) of side-effects. An increasing number of anti-seizure medications (ASM) was associated with a reduced risk (OR = 0.524) of stopping LCM. To conclude, LCM is an effective ASM with acceptable side-effects in children with drug-resistant epilepsy.

## Highlights

• The retention rate of lacosamide at 3, 12 and 24 months was 89.9%, 68.4% and 54.4%.
• Somnolence, behaviour changes, headache and dizziness occured as side-effects.
• Sodium channel blocker as concomitant use resulted in a higher risk of side-effects.
• High number of concomitant anti-seizure medications result in continuing lacosamide.

## Introduction

Current anti-seizure treatments are effective in 70–80 % of children diagnosed with focal or generalized epilepsy; the remaining 20–30 % still experience epileptic seizures [1]. Moreover, anti-seizure medications (ASM) are known to induce major side-effects, like dizziness, drowsiness, and mental slowing, leading to discontinuation of long-term treatments and reduced quality of life [2]. There is, therefore, a need for new ASM to treat drug resistant epilepsy. Lacosamide (LCM) has recently (2017) been approved for pediatric use [3], [4]. By slowly enhancing the inactivation of voltage-dependent sodium channels, it reduces neuronal overactivity [5], [6]. On the basis of its effectiveness and tolerability, reported in the literature, LCM would seem to be a promising ASM in children (<18 years) with drug resistant epilepsy. Effectiveness, as well as tolerability of an ASM, can be measured using a variety of parameters. The International League Against Epilepsy (ILAE) has recommended the retention time or retention rate as an outcome measure of ASM success [7], [8].

Previous studies contain limited data about long-term efficacy of LCM in children with drug-resistant epilepsy and the follow-up is short (≤12 months). There is limited information available about the association between LCM failure and simultaneous use of a sodium channel blocker (SCB) in children. Information about prognostic factors for side-effects while using LCM or for discontinuation of LCM is minimal. These prognostic factors, which include, age, type and duration of epilepsy, intellectual disability and concomitant ASM use, have rarely been investigated in children with drug-resistant epilepsy.

We used real-life information to assess the long-term effectiveness (follow-up until 24 months) and side-effects of LCM in children aged < 18 years with drug-resistant epilepsy. Additionally, simultaneously use of SCB and other prognostic factors for occurrence of side-effects or discontinuation of LCM before 24 months, were quantified. Our study provides practitioners more guidance for treating drug-resistant epilepsy, based on real-life data, and, in particular, elaborates on information regarding long-term (>24 months) effects and prognostic factors for side-effects and discontinuing LCM before 24 months. The primary aim of this study was to investigate the long-term effectiveness (follow-up up to 24 months) of LCM in children aged < 18 years, based on the retention rate. Furthermore, we analysed the incidence of side-effects and predictive variables for discontinuing LCM before the end of the two-year period. In this study, lacosamide was added to concomitant ASM, because of the severe drug resistant aspect of epilepsy in these patients.

## Method

### Study design and participants

This retrospective study was carried out after obtaining approval from the ethics committee and informed consent from the parents or caregivers to use the medical data. The required information was extracted from the electronic files of patients who started a LCM treatment between March 2013 and September 2019 at Kempenhaeghe, a tertiary care epilepsy center in the Netherlands. Most of the drug resistant patients in this study used LCM as an off-label ASM, prior to its approval by the European Medicines Agency. Children included in this study were aged < 18 years, had been diagnosed with drug-resistant epilepsy (uncontrolled seizures with more than two previously prescribed AMSs), and had started LCM in the tertiary care center with a minimal follow-up time of two years ± two months. Furthermore, patients' files had to include an adequate description of the efficacy, side-effects and – if applicable – use of other ASM concomitantly with LCM.

### Study assessments

The retention rate of LCM, response rate while using LCM and the side-effects occurring during the first 24 months were analysed, as well as provoking factors for discontinuing LCM and occurrence of side-effects. The retention rate was defined as the number of patients still taking LCM during the follow-up period. Changes in other ASM were allowed while the patient was on LCM. An effective response rate was defined according to one of the following definitions: seizure freedom, >50 % reduction in seizure frequency at follow-up relative to starting LCM, qualitative report of seizure reduction and/or a seizure pattern acceptable to the patient. Patients with an ineffective response rate, according to the above-mentioned criteria, were divided into two groups: lack of efficacy or negative effect on seizure pattern (increase in seizure severity or frequency). The response- and retention rates were analysed three, twelve and twenty-four months after the start of LCM. A window of two months at follow-up was used, in case the patients’ appointments did not match the time-interval of the study.

Furthermore, the reason for discontinuation was analysed. Regarding the occurrence of side-effects, special attention was given to the period after starting LCM or changing its dosage. The dosage, time interval between starting LCM and onset of side-effects, the duration of side-effects and possible interventions leading to disappearance of adverse effects were noted.

Predictive variables for discontinuing LCM before 24 months and for side-effects were studied. The predictive values were analysed for the following baseline characteristics: gender, mean age at initiation of LCM, mean age at first seizure, mean duration of epilepsy before starting LCM, etiology of epilepsy, seizure type, number of ASM used prior to LCM, the total number of concomitant ASM at start of LCM, comorbidities, and whether or not using at least one SCB at start of LCM.

### Statistical analysis

IBM SPSS statistics version 27 was used for the statistical analysis. For the response rate, the one-sample proportion test (binominal test) was used. Retention rates were analysed by Kaplan Meier. To compare characteristics between the groups, discontinued and continued LCM, a chi-square test or Fisher’s exact test was used for qualitative variables and the Mann-Whitney *U* test for quantitative variables. The same analytical methods were used for the comparison of characteristics in patients with and without side-effects. Predictive values were analysed using a univariable binominal logistic regression model. The likelihood ratio chi-square test was applied for the goodness-of-fit tests. Statistical significance was set at p < 0.05.

## Results

### Characteristics of the participants

**Fig. 1:**
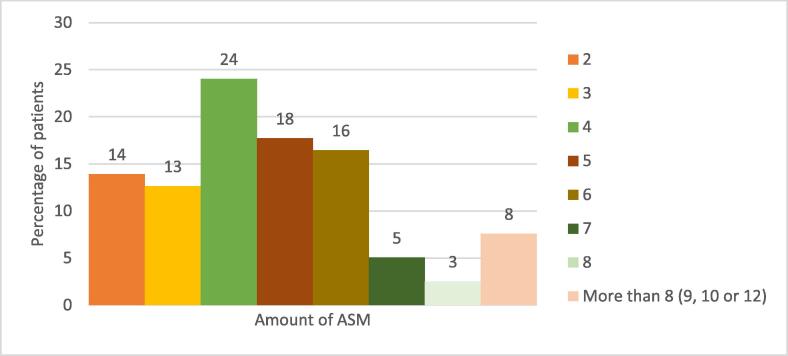
Total number of anti-seizure medications used prior to starting LCM.

**Fig. 2:**
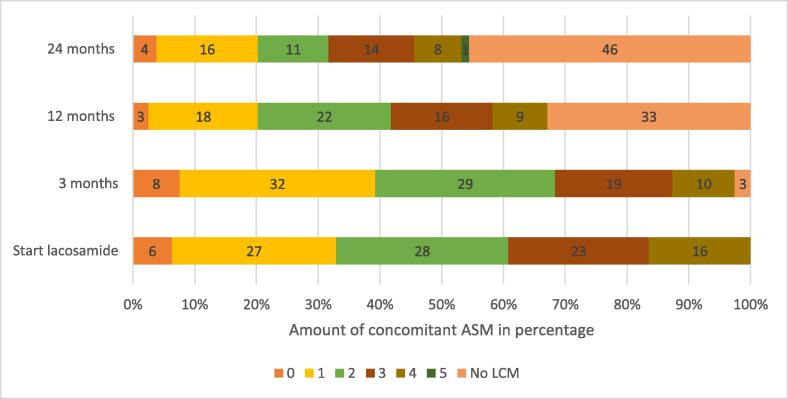
Number of concomitant ASM used at start, 3, 12 and 24 months of follow-up presented as percentage of patients at each time point.

**Fig. A1:**
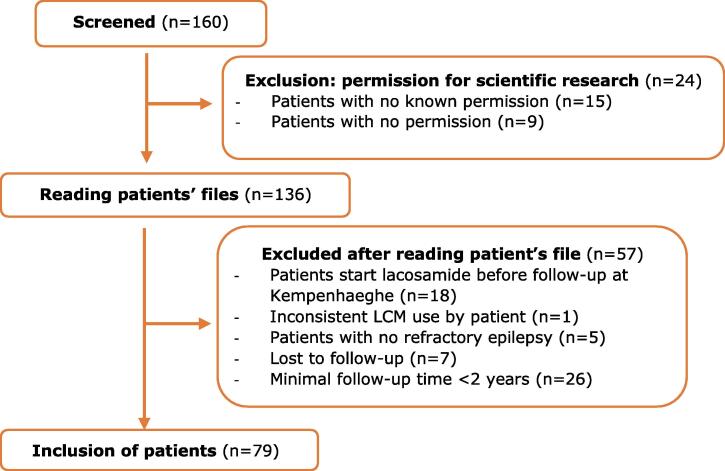
Selection of patients’ files.

**Fig. A2:**
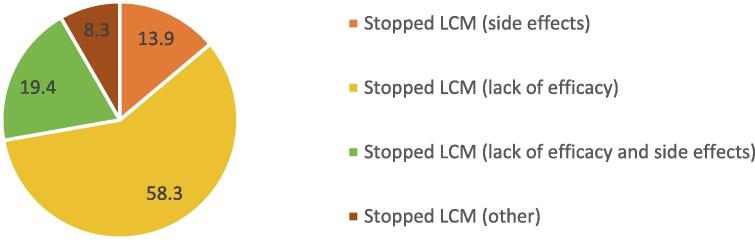
Discontinuation reason at the end of two years’ follow-up as percentage of patients (n = 36).

**Fig. A3:**
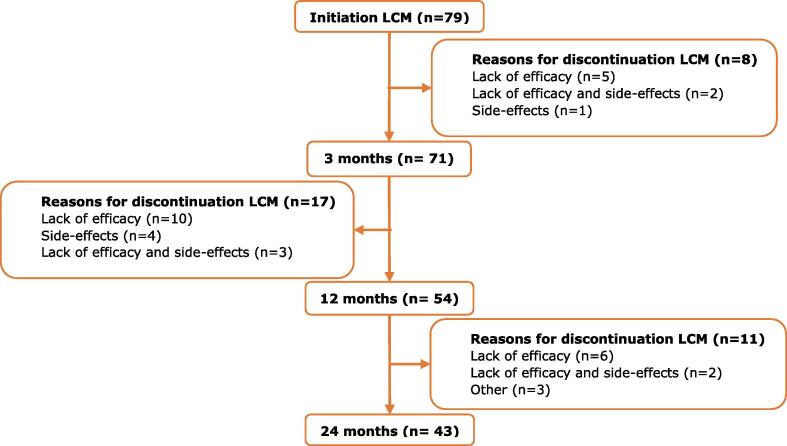
Reason for termination presented at the time-interval.

Of 160 patients’ files, 79 files were included in this study. Twenty-four files were excluded because of no or unknown informed consent. Eighteen patients started LCM before referral to the tertiary center, but implementation was not well documented. There was no adequate follow-up of seven patients, because they switched hospitals during LCM treatment or started evaluation for epileptic surgery with frequent medication changes. In twenty-six patients, follow-up was not minimally-two years. Five patients had no drug-resistant epilepsy and one patient had inconsistent use of LCM. The reasons to exclude these patient files are shown in Fig. A1, Fig. A2, Fig. A3. The characteristics of participants at baseline are shown in Table 1. Most of the patients were female (54.4 %). The patients were aged < 18 years with a mean age of 9.8 ± 3.3 years. LCM was most frequently used as adjunctive therapy (93.7 %); the LCM was added to the current ASM of the patient. Mean duration of epilepsy before starting LCM was 6.3 ± 3.4 years. The etiology of epilepsy was largely unknown (41.8 %) or of genetic origin (25.3 %). Most of the patients (68.4 %) had a focal or multi-focal primary seizure semiology and a motoric expression (70.9 %). The most common comorbidity seen was intellectual disability (IQ < 70) with an occurrence of 51.9 %. The IQ was determined on psychological tests or by estimation of the caregiver based on school reports or functioning of the child. The prevalence of diagnosed attention deficit hyperactivity disorder (ADHD) was 12.7 % and diagnosed autism spectrum disorder (ASD) was 15.2 %. As shown in Fig. 1, most patients (24.1 %) had tried four different kinds of ASM prior to starting LCM. As shown in Fig. 2, at the start of LCM treatment, the vast majority of patients (>70 %) was using two or more ASM, indicating the complexity of epilepsy in the study population.

**Table 1:** Patient characteristics.

Characteristics (n = 79)	Value
Female, *n (%)*	43 (54.4)
Mean age at start LCM, *year* ± SD	9.8 ± 3.3
*- Toddler (2-4y), n*	6
*- Child (5-11y), n*	47
*- Adolescent (12-16y), n*	26

Adjunctive lacosamide, *n (%)*	74 (93.7)
Total amount of concomitant SCB at start of LCM in patients n, (%)	
*- 0*	36 (45.6)
*- 1*	38 (48.1)
*- 2*	5 (6.3)
	
Mean age at first seizure, *years* ± *SD*	3.5 ± 3.0
Mean duration of epilepsy before starting LCM, *years* ± *SD*	6.3 ± 3.4
Mean seizure frequency per month when starting LCM, number ± *SD*	50 ± 95
Etiology of epilepsy, *n (%)*	
*- Structural*	18 (22.8)
*- Genetic*	20 (25.3)
*- Infection*	8 (10.1)
- Unknown*	33 (41.8)
	
Primary seizure type**, *n (%)*	
*- Focal*	54 (68.4)
*- Generalized*	11 (13.9)
*- Both*	13 (16.5)
*- Unknown*	1 (1.3)
	
Motor-onset seizure, *n (%)*	56 (70.9)
Nonmotor-onset seizure, *n (%)*	5 (6.3)
Both, *n (%)*	18 (22.8)
	
Awareness, *n (%)*	
*- Intact*	16 (20.3)
*- Impaired*	30 (38.0)
*- Unknown*	33 (41.8)
	
Intellectual disability (IQ < 70)^***^, *n (%)*	
*- Yes*	41 (51.9)
*- No*	33 (41.8)
*- Unknown*	5 (6.3)
	
Diagnosed ADHD, *n (%)*	10 (12.7)
Diagnosed ASD, *n (%)*	12 (15.2)

* based on full etiological screening (e.g. imaging/genetic testing/metabolic testing).

**ILAE seizure classification 2017 [9].

**Determined by neurocognitive tests or estimated by the child neurologist.

LCM = lacosamide.

### Retention rate

**Fig. 3:**
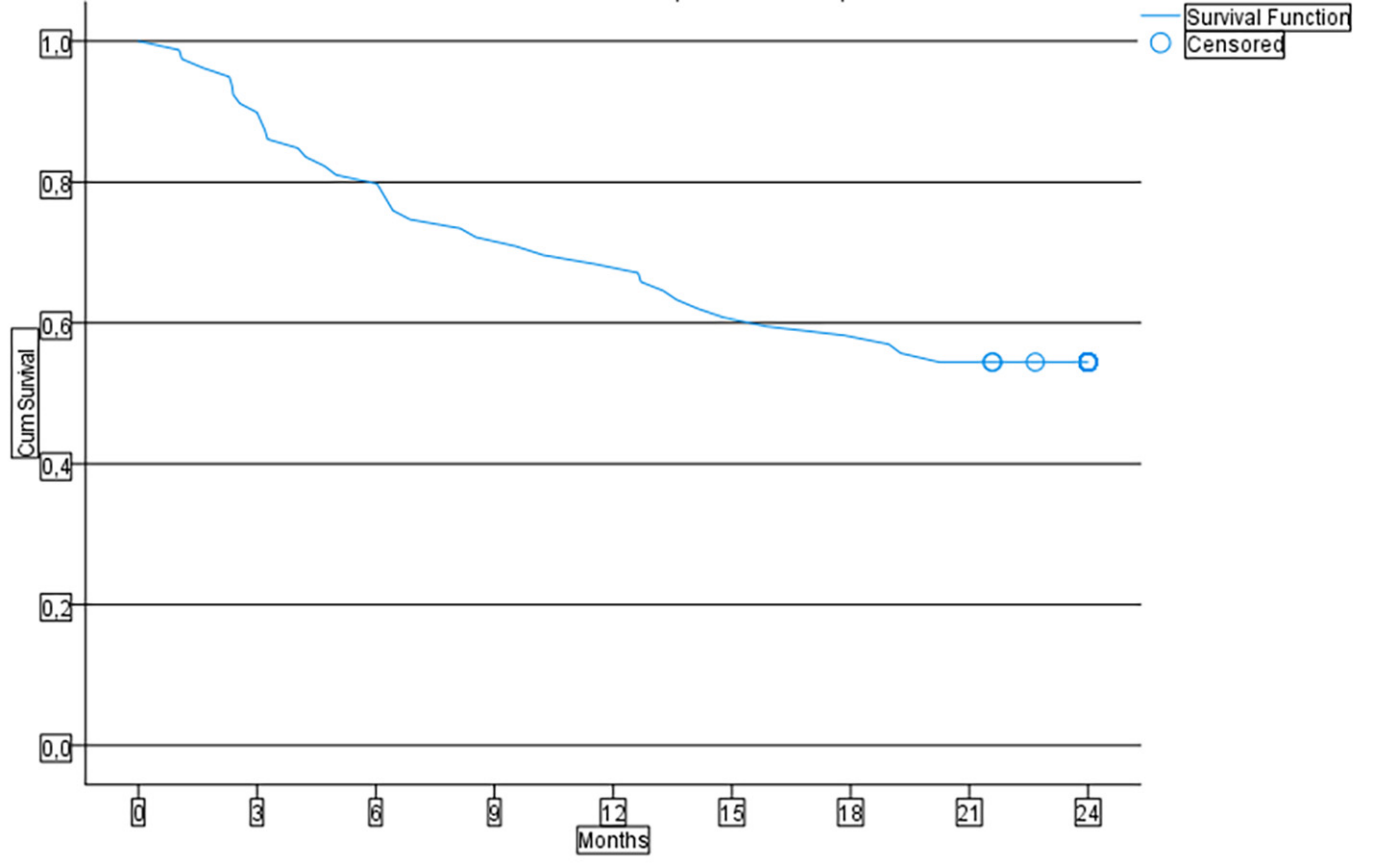
LCM retention rate with a follow-up time of 24 months.

The retention rate of LCM in all patients who started LCM treatment (n = 79) is represented in Fig. 3 by a Kaplan Meier curve. The figure shows a gradual decrease in the retention rate over the study period. At 24 months, 54.4 % of the patients were still using LCM. The decrease in retention rate seemed to be slightly larger within the first six months compared to the period between six and twenty months, after which a plateau was reached. The mean retention time was 16.83 (14.898–18.753) months. Retention at 3, 12 and 24 months was 89.9 % (n = 71), 68.4 % (n = 54) and 54.4 % (n = 43), respectively.

### Effectiveness

As shown in Table 2, LCM was effective in 60.5 %, 67.9 % and 71.4 % of the participants who were still using LCM at 3, 12 and 24 months, respectively.

**Table 2:** The percentage of effectiveness in participants using lacosamide at 3,12, and 24 months.

**Time interval**	**P-value**	**Estimate percentage of patients with an effective response (95 % CI)**	**n**
3 months	<0.001	60.5 (48.6–71.6)	76
12 months	<0.001	67.9 (53.7–80.1)	53
24 months	<0.001	71.4 (55.4–84.3)	42

### Reasons for discontinuation

The analysis of reasons for discontinuation was based on data from 79 patients. Within two years, 36 (46.8 %) patients stopped using LCM, lack of efficacy being the reason most frequently reported by 21 (58.3 %) patients. Of these 21 patients, six (7.6 %) stopped LCM (partly) because of a negative effect on seizure severity; seven (19.4 %) because of both lack of efficacy and side-effects, and five (13.9 %) because of side-effects. Three (8.3 %) patients stopped due to other reasons, such as no seizures in the past two years, polypharmacy or successful epileptic surgery. Forty-three patients were still using LCM at 24 months; in twelve of them (30.2 %), it was not effective. In eight of these twelve patients, variation in the response to LCM was found during follow-up. In two patients, the non-effective response to LCM might have been due to polypharmacy and changes in concomitant ASM.

The reasons for discontinuation at the end of follow-up are presented in appendices 2–3.

### Side-effects

Adequate description of side-effects was available in 77 patients’ files. Because of inadequate description of the side-effects, two (of 79) patients’ files were excluded from this analysis.

As shown in Table 3, side-effects occurred in 39 (50.6 %) participants, most experiencing more than one. Fifteen patients (19.5 %) stopped LCM (partly) because of side-effects, somnolence (18.2 %), behavioral changes (15.6 %), headache (9.1 %), and dizziness (9.1 %) being the most common. Other side-effects included gastro-intestinal issues (7.8 %), sadness (7.8 %), short memory problems (5.2 %), motion problems (3.9 %), diplopia (3.9 %), blurry vision (2.6 %), atrioventricular block (1.3 %) and respiratory problems (1.3 %). The time interval between starting LCM and appearance of side-effects was 3.5 ± 5.6 months. Most side-effects lasted 2.8 ± 3.4 months, after which they disappeared spontaneously (33.3 %), or because the patient stopped LCM (38.5 %) or lowered the dosage (33.3 %).

**Table 3:** Side-effects of LCM use which occurred during study period.

**Side-effects**	**Value**	**Mean dosage in mg/kg (SD)**
Number of patients with side-effects*, n (%)*	39 (50.6)	5.5 (2.8)
Somnolence	14 (18.2)	5.3 (3.3)
Behavioral	12 (15.6)	4.7 (2.6)
Dizziness	7 (9.1)	7.4 (2.4)
Headache	7 (9.1)	5.3 (2.5)
Gastro-intestinal	6 (7.8)	6.2 (2.2)
Sadness*	6 (7.8)	5.6 (2.9)
Short memory	4 (5.2)	3.2 (1.4)
Motion**	3 (3.9)	9.7 (2.3)
Diplopia	3 (3.9)	6.3 (2.5)
Blurry vision	2 (2.6)	9.7 (1.7)
Cardiovascular (AV-block)	1 (1.3)	5.6
Respiratory	1 (1.3)	12.3
Mean time-interval between starting LCM and appearance of side-effects, *in months ± SD*	3.5 ± 5.6	
Mean duration of side-effects, *in months ± SD*	2.8 ± 3.4	
Reason why side-effects disappeared, *number of patients (%)*		
Stopping LCM	15 (38.5)	
Side-effects disappeared spontaneously after a short time	13 (33.3)	
Lowering dosage of LCM	13 (33.3)	

*Sadness, crying or feelings of depression.

** Tremor or a rigid movement pattern.

### Predictive variables

Patients’ characteristics were compared to evaluate predictive variables for both discontinuing LCM before 24 months and the chance of side-effects occurring. Appendix 5, Appendix 5, Appendix 6 presents a comparison of the baseline characteristics of patients who continued and those who stopped LCM before 24 months. Five patients continued LCM up to 24 months although they had a non-effective response at three months. Appendix 5 shows the use of concomitant ASM in the two groups.

All logistic regression models represented the database, determined by the likelihood ratio chi-square test. A statistically significant difference between the groups was found in the mean number of concomitant ASM at the start of LCM (p = 0.003) and response at three months (p = 0.000). An increasing number of ASM was associated with a reduced risk (OR = 0.524) of stopping LCM at two years. If LCM was not effective at 3 months, it was associated with an increased risk (OR = 17.760) of stopping its use after two years.

The characteristics of patients with and without side-effects are shown in appendix 6. The difference between the two groups was only statistically significant in a number of patients with or without a concomitant SCB (p = 0.004), etiology of epilepsy (p = 0.021) and diagnosed ADHD (0.042). Using ≥ 1 sodium channel blocker was associated with an increased risk (OR = 4.038, CI 1.168–13.958) of side-effects. The likelihood of side-effects occurring was less (OR = 0.154, CI 0.036-0.651) in cases of epilepsy caused by structural etiology than in epilepsy with a genetic etiology. The variable ADHD had no statistical significance for the risk of side-effects.

## Discussion

Our primary goal was to evaluate the effect of LCM in children aged < 18 with drug-resistant epilepsy, paying special attention to the long-term effectiveness (follow-up to 24 months), and to give clinicians a guideline for treating these patients. Furthermore, we analysed the side-effects and predictive variables for discontinuing LCM before two years. We conclude that LCM proved to be an effective ASM with acceptable side-effects in children with drug-resistant epilepsy, based on retention rate, response rate and reason for discontinuation of the drug. As using ≥ 1 SCB at the start of LCM is associated with an increased risk of side-effects, clinicians can consider discontinuation of the concomitant SCB ASM when starting the LCM. Moreover, if LCM was not effective at 3 months, it was associated with an increased risk of stopping LCM use before/at two years. And so, if the LCM is not effective at 3 months, discontinuing LCM treatment can be considered.

### Retention rate

A retention rate reflects combined information about efficacy, quality of life, tolerability and safety and does not require a prospective baseline. Moreover, it encompasses both clinical outcomes and patient preferences [8]. Limited studies of long-term (>12 months) retention rates in children with drug resistant epilepsy are available. We found a retention rate of 89.9 %, 68.4 % and 54.4 % at 3, 12 and 24 months respectively. These results show that, in children with drug-resistant epilepsy, the willingness to continue LCM is high. A similar retention rate to the current study at 12 months was found in two retrospective studies of children with drug-resistant epilepsy in a clinical setting (72.8 % and 65 %) [10], [11]. On the other hand, two retrospective studies found a lower retention rate in participants who used LCM as an adjunctive therapy. Retention rates of 44.7 % and 25.6 % were found at 12 and 24 months in a cohort study of children and adolescents with drug-resistant focal and generalized epilepsy [12]. Another study found retention rates of, respectively, 74.4 % at 6 months, 47.7 % at 12 months, 27.9 % at 24 months, 18.0 % at 48 months, and 8.2 % at 72 months in children < 21 years [13]. The contradictive retention rates can be explained by differences in definition. In our study, changes in concomitant ASM were allowed while using LCM. In the other two studies, the retention rate was defined as using LCM without changes in concomitant ASM; therefore, these results were less compatible with real-life data. Although it is more difficult to analyze pure effects of LCM, allowing changes in concomitant medication during use of LCM reflects daily clinical practice. Remarkably, we found that some patients continued to use LCM, even though it was not effective. Some caregivers may have preferred to continue the medication because discontinuation carried a risk of destabilizing the stable but suboptimal seizure frequency. Another reason might be that in this difficult-to-treat population, a less optimal balance between efficacy and side-effects is accepted. To continue an ASM without any efficacy should, however, be prevented.

### Effective response

The definition of an effective response differs between the current study and published studies; hence no detailed comparison of the results can be made. Besides seizure freedom and >50 % reduction in seizure frequency, we also defined qualitative outcome measures of seizure reduction and/or a seizure frequency acceptable to the patient as effective responses. The latter definition is currently applied (more frequently) in daily clinical practice. We found an effective response rate of 60.5 %, 67.9 % and 71.4 % of patients still using LCM at 3, 12 and 24 months, respectively.

The ineffectiveness at 12 or 24 months can possibly be explained by the capricious evaluation of drug-resistant epilepsy, the many changes in concomitant ASM, no acceptable seizure frequency according to the patient and/or development of tolerance [14], [15]. Furthermore, the rate of an effective response depends on many factors: the individualized care and subscribing strategy for the patient, the changes in concomitant ASM use, and alternative treatment to conventional ASM [16], [17]. Also, a slow titration interval and starting LCM without a steady base of concomitant ASM, can influence the outcome in the short-term.

### Reason for discontinuation

Both in the literature and the current study, lack of efficacy was the main reason for discontinuing LCM (58.3 %)[11], [18]. In a retrospective study of 107 patients aged < 16 years with drug-resistant epilepsy, 35 % of the patients stopped using LCM. Of those patients 66 % stopped because of ineffectiveness and 18 % stopped because of side-effects [11]. Although in two patients in our study the response to LCM was not satisfactory at 12 months, LCM use was continued until 24 months, possibly because of the fear of risking destabilization of the stable but suboptimal seizure pattern.

### Side-effects

The current study observed side-effects of LCM similar to those found in other published studies. A retrospective study of 223 patients, aged < 21 years, with drug-resistant epilepsy, observed drowsiness in 35 (16 %), behavioral or mood disturbance in 24 (11 %), dizziness in 22 (10 %), gastrointestinal upset or nausea in 18 (8 %), rash in 6 (3 %), motor symptoms/ataxia in 11 (5 %), and cognitive side-effects in 5 (2 %) to be the most common side-effects [12]. As in our study, a prospective study of 30 months found that 50.6 % of the children with drug-resistant epilepsy experienced side-effects [19]. Our reported side-effects were mild to moderate in intensity, did not result in discontinuation of treatment, and in 66.6 % of the patients, the side-effects disappeared even though LCM was continued. Hence it was considered safe to use in daily clinical practice. The wide variation in the time interval of the occurring side-effects (3.5 ± 5.6 months) and duration (2.8 ± 3.4 months) can be explained by the retrospective aspect.

### Predictive values for continuing or stopping LCM before 24 months

In contrast to reports in the literature, the current study showed an association between an increasing number of concomitant ASM at the start of LCM and a reduced risk of stopping LCM at two years (OR = 0.524). A previous study in children with and without drug-resistant epilepsy, showed higher response rates with fewer concomitant ASM [10]. In general, any ASM after the first or second has diminishing efficacy (45.7 % seizure-free at the first ASM regime, compared to 14.0 % at the sixth regime) [20]. As mentioned in the paragraph on retention, a probable explanation for our findings could be that in our difficult-to-treat population, who had already tried many ASM, a less optimal balance of efficacy and side-effects is accepted.

Our study did not show an effect of concomitant SCB on the retention time, probably due to our drug-resistant population. This is in contrast to a retrospective study of 223 patients, < 21 years, using LCM specifically, which investigated the influence of concomitant SCB use [12]. They concluded that simultaneous use of another SCB increases the likelihood of treatment failure by 85 %. In adults, there is also mixed evidence for reduced efficacy and higher adverse event rate in patients who use LCM together with a concomitant SCB. Further research is, therefore, necessary. [21], [22], [23], [24].

### Predictive values for side-effects

We found a more than four times increased risk of side-effects during simultaneous use of another SCB (OR 4.038). This is a higher risk than observed in previous studies with a similar study population, where adverse effects were almost three times more often reported in patients with concomitant SCB (65 % vs 39 %, OR = 2.89 and 48 % vs 35 %). [12], [25] Clinical practitioners have to be aware of an increased risk of adverse effects when LCM is used with another SCB. However, data are still limited and should be further analysed in a larger study population before any definite conclusions can be drawn.

### Limitations of the study

The main limitation of this study is the retrospective design and the limited number of patients.

Because of the retrospective nature, the information available in the patient files was (mainly) qualitative; no detailed information about outcomes or confounding factors had been systematically recorded. Data not mentioned or remembered by the patient or caregivers may be missing. However, as all patients started LCM in our hospital, caregivers and doctors would have been generally alert to the efficacy and side-effects of the newly started drug during the first months. Furthermore, the retention rate, a reflection of both efficacy and side-effects, was measured accurately. In most studies, an effective response was defined as a ≥ 50 % seizure reduction, which requires a homogeneous patient population with similarly high baseline seizure frequency. This is seldom observed in a drug-resistant study population [8]. We, therefore, used a different definition, as mentioned in the introduction, which is more comparable to daily clinical use. In the present study population, toddlers (2–4 years) were under-represented compared to children (5–11 years) and adolescents (12–16 years). The results may, therefore, be less representative for the youngest. In addition, the absence of external validity due to the single center bias could affect the general applicability of this study.

81 of 160 patients were excluded for various reasons: lack of informed consent (24), <2 years of follow-up (26) and started LCM before consultation in the tertiary center (18). We do not expect that these exclusions will have had an impact on our results. In addition, the results of our study are broadly similar to previously published studies, and so we expect our findings also to apply to other practices.

In conclusion, LCM is an effective ASM with an acceptable side-effect profile in children with drug resistant epilepsy. Concomitant use of more than one SCB is associated with a more than four fold increased risk of side-effects.

In the future, prospective, longitudinal, multicenter studies with a larger sample size, are needed to provide insight into the views of patients, doctors and caregivers about continuing LCM, with acceptability of cumulative side-effects, in difficult-to-treat pediatric populations on ASM polytherapy. Being aware of these considerations, whether or not justified, could optimize the treatment strategies of children with drug resistant epilepsy.

## Declaration of Competing Interest

The authors declare that they have no known competing financial interests or personal relationships that could have appeared to influence the work reported in this paper.

## Figures and Tables

**Appendix 4:** Characteristics of patients, LCM continued vs LCM stopped at 24 months.

**Characteristics of patients (n = 79)**	**Continued (n = 43)**	**Stopped (n = 36)**	**P-value**
Female, *n (%)*	19 (44.2)	23 (63.9)	0.122
Mean age at start LCM, *year* ± SD	9.7 ± 3.3	9.8 ± 3.3	0.852
Patients with adjunctive lacosamide, % (*n)*	95.3 (41)	91.7 (34)	0.655
Mean age at first seizure, *years* ± SD	3.9 ± 3.2	3.1 ± 2.7	0.294
Mean duration of epilepsy before starting LCM, years ± SD	5.9 ± 3.5	6.8 ± 3.3	0.231
Mean seizure frequency per month at baseline ± SD	57 ± 108	42 ± 75	0.344
Mean number of ASM used prior to LCM, ± SD	5 ± 2	5 ± 2	0.418
Mean number of concomitant ASM when starting LCM, ± SD	3 ± 1	2 ± 1	0.003*
Patients with ≥ 1 SCB, n (%)	24 (66.6)	19 (52.8)	0.854
Mean number of ASM at 2 years, ± SD	2 ± 1		
Etiology of epilepsy, *n (%)*			0.934
- Structural	8 (18.6)	10 (27.8)	
- Genetic	10 (23.3)	10 (27.8)	
- Infection	4 (9.3)	4 (11.1)	
- Unknown	21 (48.8)	12 (33.3)	
Primary semiology, *n (%)*			0.207
- Focal or multi-focal	28 (65.1)	27 (72.0)	
- Generalized	5 (11.6)	6 (16.7)	
- Both	10 (23.3)	3 (8.3)	
Motor onset seizure at baseline, *n (%)*	32 (74.4)	24 (66.7)	0.739
Non-motor-onset seizure, *n (%)*	2 (4.7)	3 (8.3)	
Both, *n (%)*	9 (20.9)	9 (25.0)	
Awareness during seizures at baseline, *n (%)*			0.306
- Intact	8 (23.3)	6 (16.7)	
- Impaired	14 (32.6)	16 (44.4)	
- Unknown	19 (44.2)	14 (38.9)	
Intellectual disability (IQ < 70), *n (%)*	23 (53.5)	18 (50.0)	0.927
Diagnosed ADHD, *n (%)*	5 (11.6)	5 (13.9)	0.746
Diagnosed ASD, *n (%)*	4 (9.3)	8 (22.2)	0.111
Non-effective response** at three months, *n (%)*	5 (11.6)	24 (66.7)	0.000*
Mean retention time of LCM, *months* ± SD		8.8 ± 6.1	

* Statistically significant.

** Not categorizable on a 4-point definition of effectiveness.

**Appendix 5:** Percentage of patients with concomitant ASM use when starting LCM: continued vs discontinued LCM use.

ASM used concomitantly when starting LCM	LCM continued at 24 months, n (%)	LCM stopped ≤ 24 months, n (%)
**Clobazam**	23 (53.5)	10 (27.8)
**Valproate**	15 (34.9)	8 (22.2)
**Topiramate**	10 (23.3)	4 (11.1)
**Lamotrigine**	10 (23.3)	10 (27.8)
**Levetiracetam**	8 (18.6)	10 (27.8)
**Carbamazepine**	9(20.9)	9 (25.0)
**Clonazepam**	5 (11.6)	0
**Perampanel**	6 (14.0)	1 (2.8)
**Oxcarbazepine**	3 (7.0)	3 (8.3)
**Zonisamide**	3 (7.0)	0
**Vigabatrin**	3 (7.0)	2 (5.6)
**Rufinamide**	3 (7.0)	0
**Only lacosamide**	2 (4.7)	4 (11.1)
**Brivaracetam**	2 (4.7)	0
**Phenytoin**	2 (4.7)	1 (2.8)
**Ethosuximide**	1 (2.3)	2 (5.6)
**Sultiame**	1 (2.3)	0
**Felbamate**	1 (2.3)	0
**Gabapentine**	0	1 (2.8)
**Everolimus**	0	1 (2.8)

**Appendix 6:** Characteristics of patients with or without side-effects within the first two years.

**Characteristics of patients (n = 77)**	**Patients with side-effects (n = 39)**	**Patients without side-effects (n = 38)**	**P-value**
Female, *n (%)*	23 (59.0)	18 (47.4)	0.573
Mean age at start LCM, *year* ± SD	10 ± 3.07	9.5 ± 3.6	0.395
Patients with adjunctive lacosamide, % (*n)*	94.9 (37)	92.1 (35)	0.675
Mean age at first seizure, *years* ± SD	3.3 ± 2.4	3.7 ± 3.5	0.872
Mean duration of epilepsy before start LCM, years ± SD	6.9 ± 3.6	5.8 ± 3.2	0.165
Mean number ASM previously used, ± SD	5 (2)	5 (2)	0.601
Mean number of concomitant ASM at start, ±SD	2(1)	2(1)	0.842
Patients with ≥ 1 SCB, n (%)	26 (66.7)	16 (42.11)	0.004*
Etiology of epilepsy, *n (%)*			0.021*
- Structural	4 (10.3)	13 (34.2)	
- Genetic	13 (33.3)	7 (18.4)	
- Infection	2 (5.1)	6 (15.8)	
- Unknown	20 (51.3)	12 (31.6)	
Primary semiology, *n (%)*			0.317
- Focal or multi-focal	30 (76.9)	23 (60.5)	
- Generalized	5 (12.8)	6 (15.8)	
- Both	4 (10.3)	8 (21.1)	
Motor-onset seizure, *n (%)*	27 (69.2)	29 (76.3)	0.492
Non-motor-onset seizure, *n (%)*	4 (10.3)	1 (2.6)	
Both, *n (%)*	8 (20.5)	8 (21.1)	
Awareness during seizures, *n (%)*			1
- Intact	7 (17.9)	9 (23.7)	
- Impaired	15 (38.5)	15 (39.5)	
- Unknown	17 (43.6)	14 (36.8)	
Intellectual disability (IQ < 70), *n (%)*	17 (43.6)	23 (60.5)	0.162
Diagnosed ADHD, *n (%)*	2 (5.1)	8 (21.1)	0.042*
Diagnosed ASD, *n (%)*	7 (17.9)	5 (13.2)	0.227
Continued vs stopped, *n (%)*	19 (48.7)	20 (51.3)	0.298
Effective at 3 months^1^*n (%)*	14 (36.8)	15 (39.5)	0.856

^1^Defined on 4-point scale.

* Statistically significant.

## References

[1] Berg A.T., Vickrey B.G., Testa F.M. (2006). How long does it take for epilepsy to become intractable? A prospective investigation. Ann Neurol.

[2] Walia K.S., Khan E.A., Ko D.H., Raza S.S., Khan Y.N. (2004). Side Effects of Antiepileptics— A Review. Pain Practice.

[3] UBC. Vimpat. Published 2017. Accessed November 10, 2021. https://www.ucb.com/stories-media/Press-Releases/article/UCB-s-anti-epileptic-drug-VIMPAT-lacosamide-receives-EU-approval-for-paediatric-use.

[4] Vimpat | European Medicines Agency. Accessed May 2, 2022. https://www.ema.europa.eu/en/medicines/human/EPAR/vimpat.

[5] CHMP. Vimpat, INN-lacosamide. Accessed January 22, 2022. www.ema.europa.eu/contact.

[6] Katzung B., Masters S.B., Trevor A.J. (2014).

[7] Chadwick D., Beghi E., Callaghan N. (1998). Considerations on Designing Clinical Trials to Evaluate the Place of New Antiepileptic Drugs in the Treatment of Newly Diagnosed and Chronic Patients with Epilepsy. Epilepsia.

[8] Ben-Menachem E., Sander J.W., Privitera M., Gilliam F. (2010). Measuring outcomes of treatment with antiepileptic drugs in clinical trials. Epilepsy Behav.

[9] Fisher R.S., Cross J.H., Souza C.D. (2017). Instruction manual for the ILAE 2017 operational classification of seizure types. Epilepsia.

[10] Sanmartí-Vilaplana F., Díaz-Gómez A. (2018). The effectiveness and safety of lacosamide in children with epilepsy in a clinical practice setting. Epilepsy Behav.

[11] Rüegger A.D., Freeman J.L., Harvey A.S. (2019). Lacosamide in children with drug-resistant epilepsy. J Paediatr Child Health.

[12] McGinnis E., Kessler S. (2016). Lacosamide use in children with epilepsy: Retention rate and effect of concomitant sodium channel blockers in a large cohort. Epilepsia.

[13] Rosati A, Lucrezia Ilvento |, Rizzi R, et al. Long-term efficacy of add-on lacosamide treatment in children and adolescents with refractory epilepsies: A single-center observational study. Published online 2018. 10.1111/epi.14071. 10.1111/epi.14071 29663335

[14] Woldman W., Cook M.J., Terry J.R. (2019). Brief Communication Evolving dynamic networks: An underlying mechanism of drug resistance in epilepsy?. Epilepsy Behav.

[15] Tolerance and the Honeymoon Effect | Epilepsy Foundation. Accessed May 14, 2022. https://www.epilepsy.com/stories/tolerance-and-honeymoon-effect.

[16] Kneen R., Appleton R. (2006). Alternative approaches to conventional antiepileptic drugs in the management of paediatric epilepsy. Arch Dis Child.

[17] Drug-Resistant Epilepsy | Epilepsy Foundation. Accessed January 26, 2022. https://www.epilepsy.com/learn/drug-resistant-epilepsy.

[18] Aneja S., Sharma S. (2013). Newer anti-epileptic drugs. Indian Pediatr.

[19] Pasha I., Kamate M., Didagi S.K. (2014). Efficacy and tolerability of lacosamide as an adjunctive therapy in children with refractory partial epilepsy. Pediatr Neurol.

[20] Chen Z., Brodie M.J., Liew D., Kwan P. (2018). Treatment outcomes in patients with newly diagnosed epilepsy treated with established and new antiepileptic drugs a 30-year longitudinal cohort study. JAMA Neurol.

[21] Sake J.K., Hebert D., Isojrvi J. (2010). A pooled analysis of lacosamide clinical trial data grouped by mechanism of action of concomitant antiepileptic drugs. CNS Drugs.

[22] Novy J., Patsalos P.N., Sander J.W., Sisodiya S.M. (2011). Lacosamide neurotoxicity associated with concomitant use of sodium channel-blocking antiepileptic drugs: a pharmacodynamic interaction?. Epilepsy Behav.

[23] Foldvary-Schaefer N., Fong J.S., Morrison S., Wang L., Bena J. (2016). Lacosamide tolerability in adult patients with partial-onset seizures: Impact of planned reduction and mechanism of action of concomitant antiepileptic drugs. Epilepsy Behav.

[24] Villanueva V., López-Gomáriz E., López-Trigo J. (2012). Rational polytherapy with lacosamide in clinical practice: results of a Spanish cohort analysis RELACOVA. Epilepsy Behav.

[25] Kleist A., Kerling F., Hamer H., Winterholler M. (2019). Lacosamide in patients with intellectual disability and refractory epilepsy. Acta Neurol Belg.

